# A case of giant retroperitoneal ganglioneuroma encasing major vascular structures

**DOI:** 10.1093/jscr/rjaf1034

**Published:** 2026-01-07

**Authors:** Lei Xiao, Guanqiang Li, Bo Hu, Yuan Sun, Xicheng Zhang, Xianchen Huang

**Affiliations:** Department of Vascular Surgery and Intervention, The Fourth Affiliated Hospital of Soochow University, Suzhou 215123, China; Medical College of Soochow University, Suzhou 215123, China; Department of Vascular Surgery and Intervention, The Fourth Affiliated Hospital of Soochow University, Suzhou 215123, China; Department of Vascular Surgery and Intervention, The Fourth Affiliated Hospital of Soochow University, Suzhou 215123, China; Department of Vascular Surgery and Intervention, The Fourth Affiliated Hospital of Soochow University, Suzhou 215123, China; Department of Vascular Surgery and Intervention, The Fourth Affiliated Hospital of Soochow University, Suzhou 215123, China; Department of Vascular Surgery and Intervention, The Fourth Affiliated Hospital of Soochow University, Suzhou 215123, China

**Keywords:** ganglioneuroma, retroperitoneal mass, vascular encasement, surgical resection

## Abstract

Ganglioneuroma (GN) is a rare benign tumor arising from sympathetic ganglion cells, often located in the retroperitoneum. We report a case of a 33-year-old woman presenting with intermittent low back pain for 2 months. Imaging revealed a large retroperitoneal mass (24 × 36 × 68 mm) encasing the inferior vena cava (IVC) and right renal vein, with a high suspicion for GN or lymphangioma. Surgical exploration via laparotomy confirmed a tough, adherent mass (5 × 8 × 10 cm) intimately associated with the IVC and renal vessels. Complete resection with vascular repair was performed. Pathology confirmed mature ganglion cells positive for SOX10 and S100, consistent with GN. The patient recovered uneventfully and remained well at 4-month follow-up.

## Introduction

Ganglioneuroma (GN) is a rare benign tumor derived from neural crest cells of the sympathetic ganglia, with an estimated incidence of 0.1%–0.2% among all retroperitoneal neoplasms [1]. Predominantly affecting young adults, GN often presents asymptomatically until achieving significant size, leading to delayed diagnosis and potential complications from mass effect or vascular encasement [2]. Retroperitoneal GN frequently mimics more common entities like lymphangioma or sarcoma on imaging, complicating preoperative planning, particularly when encasing major vessels such as the inferior vena cava (IVC) or renal veins—necessitating multidisciplinary approaches for safe resection [3].

Here, we report a case of a 33-year-old woman with a giant retroperitoneal GN intimately encasing the IVC and right renal vessels, managed via open en bloc resection with vascular reconstruction. This case emphasizes that even emerging hospitals must prioritize imaging reporting and multidisciplinary collaboration to actively manage patients with retroperitoneal masses encasing major blood vessels.

## Case report

A 33-year-old woman was admitted due to ‘intermittent low back pain for 2 months.’ She denied a history of hypertension, diabetes, or familial hereditary tumors. Physical examination revealed: alert and oriented, with stable vital signs. The abdomen was soft and flat, without superficial lymphadenopathy or superficial varicosities; no tenderness, rebound tenderness, or peritoneal irritation signs; no palpable abdominal mass; liver and spleen not palpable below the costal margin; negative renal angle percussion pain bilaterally; negative shifting dullness; and normal bowel sounds.

**Figure 1:**
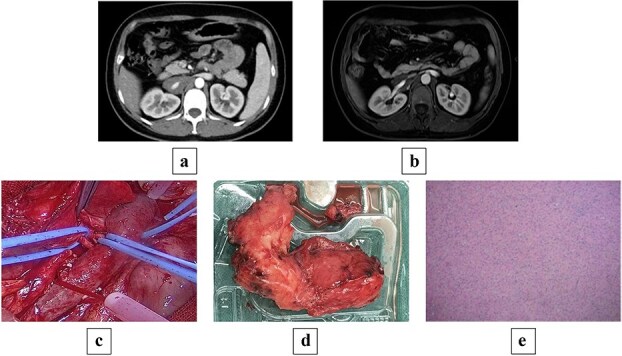
(a) Preoperative contrast-enhanced CT shows a mass-like soft tissue density shadow at the right renal hilum and adjacent to the IVC, with uniform density accompanied by punctate dense shadows at the posterior margin, measuring ~24 × 34 × 70 mm, encasing the IVC and right renal artery and vein, with partially unclear boundaries at the horizontal portion of the duodenum. (b) Preoperative MRI shows a mass-like soft tissue signal shadow at the right renal hilum and adjacent to the IVC; post-contrast scan reveals mild enhancement at the edges of the lesion, which encases the IVC and right renal artery and vein. (c) Intraoperative view of the tumor encasing the renal artery and IVC. (d) Gross pathology specimen of the resected irregular tissue fragment (8 × 6 × 2.5 cm) with gray–white cut surface, firm consistency, and attached portion of adrenal gland (2 × 1.5 × 0.3 cm). (e) Histopathological micrograph revealing mature ganglion cells with large, round nuclei and prominent nucleoli (hematoxylin and eosin stain; original magnification ×200).

Contrast-enhanced computed tomography (CT) on admission showed an occupying lesion at the right renal hilum and adjacent to the IVC, with clear borders, uniformly low density, and heterogeneous progressive enhancement post-contrast, suggestive of a possible lymphangioma (Fig. 1a). Subsequent magnetic resonance imaging (MRI) with plain and contrast-enhanced sequences revealed a right retroperitoneal mass measuring ~24 × 36 × 68 mm. On T1WI, it appeared as a uniformly low signal; on T2WI, as heterogeneous high signal. Post-contrast, it showed clear borders with heterogeneous enhancement. The lesion encased the IVC and right renal vein in an "encasing" growth pattern, without evident vascular lumen invasion or stenosis, favoring GN or lymphangioma (Fig. 1b). Endocrine evaluation, including plasma renin activity, upright posture direct renin, and aldosterone levels, showed no significant abnormalities.

Following multidisciplinary discussion (MDT), open abdominal exploration was performed. Intraoperatively, a mass was identified anterior to the IVC, encasing the IVC and right renal artery and vein, with adhesions to the vessels lacking a clear plane. The mass was firm, measuring ~5 × 8 × 10 cm. Retroperitoneal mass resection was conducted, with temporary occlusion of the bilateral renal arteries and veins (Fig. 1c). During dissection, the tumor portion had completely encased the renal vein wall, and no clear structural layer could be identified for dissection; the risk of forceful separation was high, prompting a shift to an ‘en bloc resection’ strategy, with minimal resection along the outer edge of the vein wall (~2–3 cm). Adherent portions to the renal vein were excised en bloc with the venous wall, followed by renal arteriovenous repair, resulting in blood loss of ~200 ml throughout the process.

Postoperative pathology: One irregular tissue fragment (retroperitoneal mass) measuring 8 × 6 × 2.5 cm, with gray–white cut surface and firm consistency, attached to a portion of adrenal gland measuring 2 × 1.5 × 0.3 cm (Fig. 1d). Immunohistochemistry: AE1/AE3(−), SOX10(+), S100(+), Ki67(<2%+), Melan A(−), Syn(+), CgA(+), PAX8(−), Inhibin(−). Mature ganglion cells were observed, scattered with large, round nuclei and prominent nucleoli, consistent with GN (Fig. 1e).

Postoperatively, the patient received anti-inflammatory, acid-suppressive, and anticoagulant therapy, with good recovery and discharge. Follow-up at over 4 months showed the patient in good condition, with imaging negative for residual or recurrent disease.

## Discussion

GN is a benign tumor originating from sympathetic ganglia, typically arising from neural crest cells [4]. Although extremely rare, GN grows slowly with low malignant potential, often leading to delayed diagnosis. While most GNs are benign, a minority carry a risk of malignant transformation into ganglioneuroblastoma or neuroblastoma, underscoring the importance of early diagnosis and treatment, particularly in cases with transformation potential.

Clinical manifestations of GN are often insidious, with nonspecific early symptoms. Symptoms typically emerge only when the tumor reaches a significant size, most commonly presenting as abdominal mass, pain, discomfort, or gastrointestinal symptoms [5]. Larger tumors tend to produce more pronounced symptoms. Due to the lack of specificity, GN is frequently diagnosed after exclusion of more common tumors.

Imaging is pivotal for GN diagnosis. CT and MRI are the primary modalities, delineating tumor size, location, and relationships to adjacent structures. CT typically shows a uniform low-density mass with heterogeneous progressive enhancement post-contrast; MRI reveals heterogeneous high signal on T2WI and uniform low signal on T1WI [6]. Notably, GN often encases major vessels or adjacent organs, mimicking other retroperitoneal tumors such as lymphangioma or adrenal tumors.

In this case, preoperative imaging suggested GN or lymphangioma, but comprehensive evaluation and detailed analysis favored GN. The mass was retroperitoneal, with clear borders, uniformly low density, progressive enhancement, and classic “vascular encasement” without lumen invasion or compression—highly consistent with reported retroperitoneal GN features: slow, benign growth encasing vessels (e.g. IVC or renal vessels) without wall disruption, and T2WI high signal indicating neural fibers and cystic components [7]. Lymphangioma, a common differential for retroperitoneal cystic masses, may appear as well-defined, multicystic low-density lesions with mild enhancement, particularly at the renal hilum, but GN more often shows neural bundle-like structures, while lymphangioma features pure cystic changes. Endocrine tests (renin, aldosterone) ruled out functional lesions, supporting non-adrenal origin. Thus, meticulous imaging analysis is crucial for GN diagnosis.

Although percutaneous biopsy is valuable, it was deemed risky here due to proximity to major vessels and nerves with tight adhesions. For masses near critical structures, intraoperative excision for pathology is often preferred, as in this case, based on history and imaging [8].

Surgical resection is the mainstay of GN treatment, aiming for complete excision to prevent recurrence. For retroperitoneal tumors encasing vessels, open surgery is safer than laparoscopy, ensuring completeness and minimizing vascular injury. Laparoscopy offers minimal invasiveness but is less suitable for vessel-encasing tumors [9].

As an emerging hospital, our department recognizes the complexity of retroperitoneal mass management, involving major vessels and multiple organs. We have bolstered subspecialty development via MDT collaboration among vascular surgery, endocrinology, radiology, and pathology, incorporating domestic and international guidelines and literature. This case's precise resection exemplifies our team's rapid growth and clinical innovation, offering valuable insights for similar cases.

GN has a low recurrence rate and favorable prognosis, typically without need for adjuvant chemotherapy or radiotherapy. If visceral arteries or the abdominal aorta are injured, prompt revascularization is essential. Postoperative surveillance monitors for recurrence or complications.

In summary, GN is a rare benign tumor with slow growth and low malignant risk. Lacking specific symptoms, diagnosis relies on imaging and pathology. Surgical resection is preferred, with open approaches safer for vessel-encasing tumors. Early surgery and follow-up yield excellent outcomes.
